# Roles of Oxytocin in Stress Responses, Allostasis and Resilience

**DOI:** 10.3390/ijms23010150

**Published:** 2021-12-23

**Authors:** Yuki Takayanagi, Tatsushi Onaka

**Affiliations:** Division of Brain and Neurophysiology, Department of Physiology, Jichi Medical University, Tochigi 329-0498, Japan; tonaka@jichi.ac.jp

**Keywords:** oxytocin, stress, hypothalamus, allostasis, resilience

## Abstract

Oxytocin has been revealed to work for anxiety suppression and anti-stress as well as for psychosocial behavior and reproductive functions. Oxytocin neurons are activated by various stressful stimuli. The oxytocin receptor is widely distributed within the brain, and oxytocin that is released or diffused affects behavioral and neuroendocrine stress responses. On the other hand, there has been an increasing number of reports on the role of oxytocin in allostasis and resilience. It has been shown that oxytocin maintains homeostasis, shifts the set point for adaptation to a changing environment (allostasis) and contributes to recovery from the shifted set point by inducing active coping responses to stressful stimuli (resilience). Recent studies have suggested that oxytocin is also involved in stress-related disorders, and it has been shown in clinical trials that oxytocin provides therapeutic benefits for patients diagnosed with stress-related disorders. This review includes the latest information on the role of oxytocin in stress responses and adaptation.

## 1. Introduction

Oxytocin is primarily synthesized in magnocellular and parvocellular neurons of the paraventricular nucleus (PVN) and supraoptic nucleus (SON) of the hypothalamus. Magnocellular oxytocin neurons in the hypothalamus project to the neurohypophysis and have axon collaterals to the forebrain. Oxytocin is released from the axonal terminals in the neurohypophysis to the circulatory system, playing a pivotal role in the regulation of parturition and lactation in mammals. It is also known that parvocellular oxytocin neurons project to various brain regions and release oxytocin in the projection regions. Oxytocin is released not only from axon terminals but also from cell bodies/dendrites and possibly from axonal varicosities of magnocellular oxytocin neurons and is diffused to distal action sites. Oxytocin acts on the oxytocin receptor in various brain regions. Oxytocin neurons are activated by various stressful stimuli and release oxytocin, which modulates stress responses.

**Figure 1:**
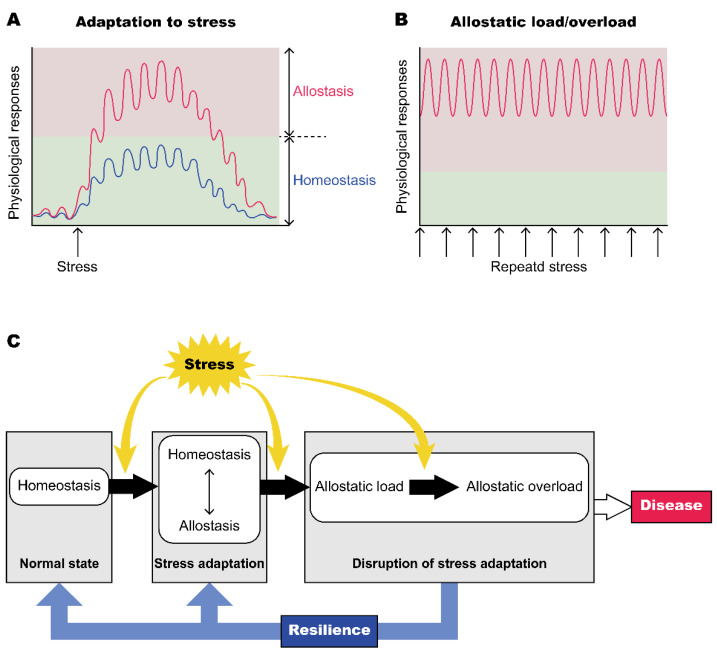
Allostasis and resilience. (**A**) Physiological responses to stressful stimuli in bodily systems including the autonomic nervous system, hormone responses, and inflammatory cytokines. Homeostasis refers to the body’s ability to maintain a stable internal milieu. Allostasis refers to the process that maintains homeostasis and to the drive of other physiological and behavioral systems by changing set points depending on changing environments. A normal allostatic response lasts for an appropriate time after stress, then returns to a normal level and ends [7]. (**B**) Allostatic load/overload is a state of chronically sustained allostasis that is induced by chronic stressful stimuli or inefficient management of allostasis. Allostatic load is also provoked by repeated normal responses over time, lack of adaptation, prolonged responses due to delayed shutdown and inadequate responses [7]. (**C**) Allostatic load or overload persistently activates physiological responses (neuroendocrine, cardiovascular and emotional responses) and accelerates the progression of various diseases. Resilience acts to restore physical and psychological conditions from a failed state of allostasis to a normal state.

Stress is a state of threatened homeostasis in response to experiences that cause physical, emotional and psychological challenges that exceed the capacity of the individual to cope. Homeostasis is defined as maintenance of an internal environment that includes physiological variables such as heart rate, blood pressure, body temperature and blood sugar within a certain narrow range [1]. Homeostasis requires monitoring of these physiological parameters that are vital for life in order to keep values constant via feedback mechanisms. Owing to homeostasis, bodily systems are protected from being out of order due to harmful events [2]. However, various physiological parameters in fact show fluctuations depending on environmental challenges, and the concept of allostasis has recently become more prevalent. Allostasis was defined by Sterling and Eyer as “achieving stability through changes” [3]. Allostasis now refers to the regulation of internal milieu through gradual changes toward new set points. It is also thought that changes of set points by allostasis lead to other physiological and behavioral systems being driven to adapt to a changing environment. Bodily systems show adaptive processes in preparation for anticipated future changes in internal or external conditions. Shifts of set points dependent on conditions and activations of emergency systems are adaptive for a short period, while shifts and activations for a long period often cause overloads in various bodily systems, resulting in lifestyle-related disorders (Figure 1) [4,5]. It is important to restore the set points to their original place and to recover normal status before organs receive irreversible damage. Resilience refers to an individual’s capacity to make a successful recovery from psychological or physical difficulties. These difficulties include family problems, interpersonal relationships, health problems, workplace load and financial stressors. Resilience helps a person bounce back and recover from these difficult experiences [6]. In other words, resilience is the ability to restore a shifted set point and emergency system to their original setting after coping with difficulties and regain homeostasis (Figure 1). Molecular and neural mechanisms underlying allostasis and resilience still remain to be clarified. The present review focuses on the role of oxytocin in the control of stress responses, allostasis and resilience.

## 2. Stress Responses and Oxytocin

### 2.1. Activation of Oxytocin Neurons and Facilitation of Oxytocin Release after Various Stressful Stimuli

Animal studies have shown that various physiological and psychological stressful stimuli, such as noxious stimuli [8], conditioned fear stimuli [9], social defeat stress [10,11,12,13,14], immobilization stress [15], shaker stress [16], forced swimming [17,18], cold stress [19], high-intensity exercise [20] and immune challenges by lipopolysaccharides [21] or interleukin-1 [22], activate oxytocin neurons and facilitate the release of oxytocin into the plasma and within the brains of mice and rats [23,24,25]. Social instability stress in adolescent female rats has been reported to decrease oxytocin immunoreactivity in the hypothalamic paraventricular nucleus [26] possibly due to oxytocin release. Synchronic oxytocin release into the plasma and within the brain has been shown during forced swimming stress [18] and shaker stress [16]. On the other hand, dissociated oxytocin release has also been reported during social defeat stress [13] in rats. 

In humans, oxytocin release after stressful stimuli has also been reported. Physical running [27,28,29,30], psychological stress such as uncontrollable noise in women [31] and a Trier social stress test that consists of a public speaking task and mental arithmetic performed in front of an audience [27,32,33] have been shown to increase plasma or salivary oxytocin concentrations. A positive correlation between plasma oxytocin concentrations and anxiety or relational distress has been shown in healthy humans [34,35,36] and in patients with a social anxiety disorder [37]. High levels of plasma oxytocin have also been reported in subjects with high depressive scores [38]. The number of oxytocin neurons [39], expression of oxytocin mRNA in the hypothalamus [40] and plasma oxytocin concentrations [41] have also been shown to be increased in depressed subjects. 

However, apparently discrepant data concerning plasma oxytocin concentrations in stressed conditions have also been reported. Exercise of short duration and high intensity has been shown not to significantly change plasma oxytocin concentrations in humans [42,43]. In rats, plasma oxytocin levels have been reported not to change after exercise [44]. Plasma or urine oxytocin concentrations have also been reported to not be significantly changed by a Trier social stress test in children [45] and in women [46,47] and by a speech stress task in women [48]. Furthermore, decreased oxytocin levels have been observed in the cerebrospinal fluid (CSF) of women with a history of child abuse [49], in the CSF of suicide attempters [50], and in the plasma of patients with post-traumatic stress disorder (PTSD) [51]. Decreased oxytocin concentrations in the blood have been found in patients with depression [52,53]. Research concerning postpartum depression has shown that plasma oxytocin levels were inversely related to depressive symptoms [54]. These differences in data might be caused by differences in subjects, species, stressful stimuli applied and experimental conditions. The interpretation of oxytocin levels in psychiatric diseases is complex. A decrease in oxytocin levels in patients with psychiatric diseases might be a cause of the development of anxiety or depression-like symptoms in these patients, and might not be a result of stressful stimuli. Considering these discrepant findings, oxytocin does not appear to be a general biomarker for psychiatric disorders.

### 2.2. Roles of Oxytocin in Stress Responses

Oxytocin regulates stress responses in the neuroendocrine system, autonomic nervous system, immune system and behaviors. In many studies using both animal and human subjects, oxytocin has been shown to reduce the activity of the hypothalamic-pituitary-adrenal (HPA) axis [55,56,57,58,59,60], regulate autonomic stress responses [19,61,62,63,64], attenuate inflammation [65,66,67] and reduce anxiety-related behaviors [23,24,68,69,70]. Oxytocin-deficient female mice, but not oxytocin-deficient male mice, have been reported to show increased anxiety-related behaviors in an elevated plus maze test [71]. A high plasma level of corticosterone after shaker stress and novel environmental stress-induced hyperthermia have been also observed in oxytocin-deficient female mice [71]. These results suggest that endogenous oxytocin reduces the activity of the HPA axis and the anxiety-related behavioral system during stress. In many studies in which the effects of oxytocin administration on anxiety-related behavior and fear conditioning were assessed, oxytocin was shown to decrease anxiety-related behavior [68,70,72], attenuate contextual fear expression and facilitate extinction in rodents [68,70]. 

Studies with human subjects have also shown anxiolytic effects of intranasally administered oxytocin [70]. Oxytocin administration and social support have been shown to decrease cortisol levels and reduce anxiety during a Trier social stress test in healthy men [73]. Intranasal oxytocin has been reported to reduce the activity of the amygdala region in response to fearful faces or fear scenes in healthy subjects [74,75,76,77,78,79]. In patients with generalized social anxiety disorder, hyper-reactivity to fearful faces in the left amygdala has been reported to be attenuated by intranasal oxytocin [80]. 

The regulation of stress responses by oxytocin is observed in a variety of social situations. Social relationships are important for maintaining both mental and physical health [81,82]. Social buffering, the attenuation of stress responses in affiliative social conditions, is partly responsible for the positive impact of social relationships. The oxytocin system has been shown to attenuate stress responses in the presence of socially affiliative conspecifics [83]. In female prairie voles, cohabitation with a male partner has been shown to reduce anxiety-related behavior and corticosterone levels in response to immobilization stress via the hypothalamic oxytocin system [84]. In humans, social buffering is associated with a single nucleotide polymorphism of the oxytocin receptor gene [85]. Some species have been shown to exhibit consolation-like behavior, which attenuates stress responses, toward distressed affiliative conspecifics. Oxytocin has also been implicated in consolation-like behavior. Prairie voles have been reported to show consolation-like allogrooming behavior towards distressed partners via the oxytocin system. Allogrooming decreases anxiety-related behaviors and corticosterone levels [86]. Mandarin voles [87] and female mice [88] have also been reported to show consolation-like allogrooming behavior towards socially defeated partners via the oxytocin system. 

Parent-infant interaction in humans, which is positively correlated with oxytocin levels in both parents and infants, has been reported to decrease salivary cortisol levels [89]. Mating, which activates the oxytocin system, has been shown to decrease anxiety-related behaviors in male rats [90]. The oxytocin receptor expressed in melanin-concentrating hormone (MCH) neurons of the lateral hypothalamus has been shown to be involved in mating-induced decreases in depressive-like behavior in male mice [91]. It is known that wound healing is impaired by psychological stress in rodents and humans. Oxytocin administration has been shown to decrease cortisol and facilitate wound healing in socially isolated hamsters, while an oxytocin receptor antagonist has been reported to delay wound healing in socially housed hamsters [92].

These findings suggest that the oxytocin system is activated in response to stress and in socially affiliative conditions, and that the oxytocin system attenuates the stress response. The oxytocin system also indirectly alleviates the stress response in distressed individuals by inducing the seeking of social support and by receipt of social support [45,73,85,93,94,95]. 

On the other hand, anxiogenic action of oxytocin has also been reported. Chronic injection of oxytocin has been shown to increase anxiety-related behavior in male mice [96] but not in female rats with high anxiety [97] or in lactating female rats [98]. Viral vector-induced overexpression of the oxytocin receptor in the lateral septum has been shown to enhance contextual fear in socially defeated mice [99]. Interestingly, chronic oxytocin administration has been reported to enhance anxiety-related behaviors in male rats via inducing alternative splicing of the hypothalamic corticotropin-releasing factor receptor 2α (*Crfr2*α) and shifting the splicing ratio from the anxiolytic membrane-bound CRFR2α form to the soluble CRFR2α form [100]. In healthy subjects, oxytocin has been shown to facilitate recognition of threatening stimuli and fearful facial expressions [101,102,103], suggesting that oxytocin seems to enhance anxiety. No significant effects of intranasal oxytocin on core symptoms in patients with social anxiety disorder and depression have also been reported [104]. The effects of oxytocin appear to vary considerably depending on the gender, social context, and mode of oxytocin administration; treatment via oxytocin administration will therefore require further in-depth research. Oxytocin also has been shown to be involved in early life stress and brain plasticity (see another review [105]).

### 2.3. Site of Action of Oxytocin with Respect to Stress Responses

The oxytocin receptor is distributed widely within the brain, which regulates stress responses, including the prefrontal cortex, limbic area, hypothalamus, raphe and medulla oblongata [106].

#### 2.3.1. Prefrontal Cortex

The oxytocin receptor in the prelimbic or infralimbic prefrontal cortex and in the anterior cingulate cortex (ACC) has been shown to have anxiolytic effects. 

Oxytocin injection (male rats) [107] and activation of oxytocin receptor-expressing neurons (male mice but not female mice) [108] in the prelimbic prefrontal cortex have been shown to decrease anxiety-related behavior. Optogenetic activation of the prelimbic prefrontal cortex oxytocin terminals projecting from the PVN oxytocin neurons has been shown to reverse paternal deprivation-induced increases in anxiety-related behavior and social avoidance in mandarin voles [109]. In the infralimbic prefrontal cortex, oxytocin has been reported to induce fear extinction [110]. Furthermore, social interaction-induced effects on contextual fear memory extinction have been shown to be mediated by infralimbic oxytocin signaling [111]. 

Chronic social defeat stress has been reported to decrease expression of oxytocin receptor mRNA in the ACC, inducing anxiety-related behaviors. Oxytocin injection into the ACC has been shown to attenuate nociceptive responses and anxiety-related behavior in animals with neuropathic pain by selectively blocking the maintenance of presynaptic long-term potentiation (LTP) but not postsynaptic LTP [112]. In humans, intranasal oxytocin has been shown to reduce activation of the dorsal part of the ACC in response to fearful faces in men, while intranasal oxytocin increased ACC activation in women [113].

#### 2.3.2. Bed Nucleus of the Stria Terminalis

The oxytocin system in the bed nucleus of the stria terminalis (BNST) has been reported to have anxiogenic effects.

Fear conditioning has also been shown to activate oxytocin neurons in the PVN and SON, and to induce oxytocin release in the dorsolateral BNST [114]. Oxytocin signaling in the dorsolateral BNST has been shown to facilitate acquisition of cued fear in fear-potentiated startle [115]. Inhibition of the oxytocin receptor in the anteromedial BNST has been reported to restore normative social approach behavior following social defeat in California mice [10]. Oxytocin neurons in the medioventral BNST, from which fibers extend into the anteromedial BNST, anterior hypothalamus and lateral hypothalamus, act on the induction of social stress-induced social anxiety behaviors including social vigilance and avoidance in California mice [116]. 

#### 2.3.3. Hypothalamus

The oxytocin receptor in the PVN has been reported to have anxiolytic effects and the oxytocin receptor in the lateral hypothalamus has been shown to modulate depressive-like behavior.

Local injection of oxytocin into the PVN of male rats [117] and female prairie voles [118] has been shown to decrease anxiety-related behavior. Oxytocin has been reported to increase the activity of GABAergic neurons in the PVN, and blockade of the GABA_A_ receptor in the PVN has been shown to eliminate the oxytocin-induced decreases in anxiety-related behavior and corticosterone levels following stress in female prairie voles [118]. Oxytocin has also been reported to decrease the activity of corticotrophin-releasing hormone (CRH) neurons in the PVN following stress in voles [118]. The oxytocin receptor is expressed in CRH neurons of the PVN, and oxytocin released by acute salt loading has been suggested to tonically inhibit PVN CRH neurons via the oxytocin receptor [119]. 

The oxytocin receptor expressed in MCH neurons in the lateral hypothalamus has also been shown to attenuate depressive-like behavior in sexually naïve females, but to augment depressive-like behavior in late postpartum female mice [91].

#### 2.3.4. Lateral Septum

The oxytocin receptor in the lateral septum has been reported to have both anxiogenic and anxiolytic effects. Apparently, the contradictory findings in these reports remain to be explained.

Oxytocin application has been reported to increase recall of negative events and stressful stimuli [102,120]. Deletion of the oxytocin receptor or injections of oxytocin antagonists in the lateral septum of male mice have been reported to decrease freezing behavior in social defeat stress-induced contextual fear and to block social aversion toward aggressive resident mice. Conversely, overexpression of the oxytocin receptor in the lateral septum has been reported to exacerbate freezing behavior in social defeat stress-induced contextual fear and social defeat stress-induced social aversion [99]. These results suggest that oxytocin in the lateral septum potentiates fear by facilitation of social fear memory. Activation of oxytocin receptor-expressing neurons in the lateral septum projecting to the horizontal diagonal band of Broca has been shown to induce anxiety-related but not depressive-like behaviors via GABA in mice, suggesting anxiogenic action of the oxytocin receptor in the lateral septum [121]. 

On the other hand, there have been studies showing that oxytocin in the lateral septum has anxiolytic effects. Oxytocin injection into the lateral septum of male mice has been reported to abolish social fear expression in a social-fear-conditioning paradigm [122]. In addition, the naturally activated oxytocin system in lactating mice has been reported to prevent social fear expression in a social-fear-conditioning paradigm, while silencing of oxytocin neurons in the PVN projecting to the lateral septum has been shown to enhance social fear in lactating female mice [123].

#### 2.3.5. Amygdala

The oxytocin receptor in the central amygdala (CeA) has been shown to have anxiolytic action in various studies. However, contradictory data have also been reported. Oxytocin in the amygdala has also been reported to facilitate the recognition of emotions, leading to modulation of stress-coping behavior.

Oxytocin is known to reduce fear and anxiety by reducing the activity of the amygdala. Oxytocin injection into the CeA has been shown to attenuate long-term isolation-induced depressive-like and anxiety-related behaviors in male mice [124] and to decrease anxiety-related behavior in female rats [125]. Activation of the CeA terminals from oxytocin neurons in the hypothalamus has been shown to decrease contextual freezing in fear-conditioned female rats [126]. Injection of an oxytocin agonist into the CeA has been reported to decrease freezing behavior in fear conditioning test. This phenomenon has been reported to be mediated by activation of oxytocin receptor-expressing GABAergic neurons in the lateral part of the CeA that inhibit neurons in the medial part of the CeA projecting to the periaqueductal gray [127]. However, mixed and contradictory data concerning the effects of oxytocin on fear conditioning in the basolateral amygdala and the CeA have also been reported (Please see other reviews for details [24,69]). 

Oxytocin in the amygdala may switch stress-coping behavior. Injection of an oxytocin receptor antagonist into the CeA of rat dams has been reported to prevent the switch from self-defense freezing behavior to offspring protective behavior in mothers in response to exposure to a threatening situation, and pups have been shown not to acquire fear learning from these mothers whose amygdala oxytocin receptor was blocked [128]. 

Oxytocin in the amygdala also may modulate stress coping behavior by facilitating recognition of emotion. Oxytocinergic projections from the PVN to the CeA are necessary for discrimination of both positive and negative emotions of unfamiliar conspecifics in male and female mice [129].

#### 2.3.6. Raphe Nucleus

The oxytocin receptor in the raphe nucleus has been shown to have anxiolytic actions. The oxytocin receptor is expressed in approximately half of the serotoninergic neurons in the raphe nucleus, and local injection of oxytocin into the median raphe decreases anxiety-related behaviors by facilitation of serotonin release, possibly via the serotonin 2A/2C receptor [72]. However, deficiency of the oxytocin receptor in serotoninergic neurons in the raphe nucleus has been shown to have no significant influence on anxiety-related behavior, but to enhance aggression only in male mice [130]. The discrepancy in results may be caused by the difference in experimental procedures. The former study showed that oxytocin-induced anxiolytic actions were blocked by a serotonin receptor antagonist. The latter study showed that mice deficient in the oxytocin receptor in serotonin transporter-expressing cells had no obvious changes in anxiety-related behavior. Lifelong deficiency in the oxytocin receptor might have induced compensatory mechanisms and, as a result, the knockout mice might have shown no obvious phenotypes.

#### 2.3.7. Medulla Oblongata

The oxytocin receptor in the dorsal motor nucleus of the vagus has been shown to reduce visceral stress responses. Activation of oxytocin neurons in the PVN projecting to the dorsal motor nucleus of the vagus has been reported to prevent the delayed gastric emptying observed following acute or chronic heterotypic stress and to increase gastric tone and motility following chronic heterotypic stress in rats [131].

## 3. Allostasis and Oxytocin

Concerning the effects of oxytocin on adaptations to stress, it has recently been pointed out that oxytocin plays a pivotal role not only in maintaining homeostasis, but also in allostasis. The concept of allostasis was proposed about 30 years ago. Allostasis is efficient adjustment by organisms to various conditions achieved by altering the set point of bodily parameters and controlling them predictively. The regulatory scheme for correcting errors through feedback mechanisms to maintain a stability is homeostasis. Regulation of changes in the set point and efficient prevention of errors by prediction and feedforward mechanisms, in combination with switching the coping strategy depending on the environment, is referred to as allostasis. Both regulation systems are important for complementary maintenance of body internal milieu. The brain plays an essential role in the prediction of future demand and in a change to an appropriate coping strategy for maintaining the bodily system [132]. 

### 3.1. Oxytocin in the Concept of Allostasis

Oxytocin has been shown not only to facilitate prosocial or socially positive behavior, but also apparently anti-social or socially negative behaviors, including aggression, depending on the situation [133]. Oxytocin has been shown to facilitate in-group trust, cooperation and conformity, but also aggression toward competing out-groups [134,135,136]. The results of those studies show that oxytocin facilitates not only pro-social behavior, but also anti-social behavior, depending on the social environment. Oxytocin decreases meal size and induces satiety in adult animals, while oxytocin induces the onset of feeding behavior at birth and maintains food intake during the postnatal period [137]. Induction of satiety by oxytocin may contribute to food sharing in group life and may be adaptive. On the other hand, initiation of food intake by oxytocin at birth is essential for survival. Thus, it is possible that the apparently opposite effects of oxytocin on food intake dependent on developmental states are both adaptive. It is likely that effects of oxytocin depend on the context and that oxytocin acts as an allostatic hormone to facilitate adaptation, integration and stability in changing environments [138]. The oxytocin system might facilitate the sensing of internal or external signaling [139] and contribute to the adjustment of set points, depending on conditions. For example, regarding the perception of social signals, oxytocin has been shown to increase the signal-to-noise ratio in olfactory signaling via exciting inhibitory neurons [140] and in auditory signaling via balancing inhibition with excitation [141]. Thus, it seems that oxytocin increases the salience of social stimuli, induces plasticity and facilitates recognition of the environmental context in order to adapt to changing environments. The oxytocin system also facilitates learning of stimulus-outcome associations and assists in future predictions to adapt bodily systems according to changing environments [138]. Oxytocin activity has been reported to weaken the decline of the subjective value of delayed reward and to resist immediate temptation impulses, leading to future-oriented behavior [142]. Oxytocin also facilitates reversal learning to increase behavioral flexibility in humans [142] and in Brown Norway rats [143]. These actions have been shown to be impaired in oxytocin receptor-deficient mice [144], suggesting that the oxytocin system is essential for the ability to learn a new strategy for behavioral flexibility and the ability to adapt to a changing environment. In humans, oxytocin administration has also been reported to increase learning performance during conditioning in fear conditioning tests, inducing rapid adaptations to frightening stimuli and augmenting fear responses in aversive contexts [145]. These results have led to the argument that oxytocin is not simply anxiolytic but induces rapid and flexible adaptation to aversive situations. Under stressful conditions, oxytocin reduces passive freezing behavior [146], increases stress-induced analgesia [147,148], and increases stress-induced oxygen consumption [23] to facilitate muscle movement for active coping behaviors.

The oxytocin system has been shown to play a role in allostasis during reproductive behavior. Oxytocin is released at the time of delivery and plays not only a pivotal role in uterine contraction in mothers, but also in analgesia in the newborns [149]. Oxytocin has been shown to increase the threshold of pain sensation in newborn rats (P0) via reduction of the depolarizing action of GABA on nociceptive neurons [150]. Oxytocin reduces intracellular chloride concentration and switches GABA function from excitation to inhibition [151]. Maternal oxytocin during delivery might also exert a neuroprotective action on fetal neurons during parturition. The shift in GABAergic actions induced by oxytocin also changes the balance of excitation and inhibition in neural circuits and constitutes an important element of allostatic systems. 

Oxytocin is also involved in allostasis during lactation. In lactating rodents, suckling stimuli from pups facilitate oxytocin release within the brain and raise the set point for the induction of anxiety in response to extrinsic factors. Oxytocin induces not only an anxiolytic action, but also evokes active coping behaviors such as maternal aggression toward intruders [152] and protective behavior to threatened signals [128] in order to defend their pups. 

### 3.2. Adaptation to Changing Environments in the Oxytocin System

The oxytocin system shows adaptive changes dependent on internal and external conditions. The activity of oxytocin systems can be plastically changed via the modulation of the intrinsic properties of oxytocin neurons themselves, and via modulation of input signals to oxytocin neurons, leading to modulation of physiological set points to adapt to changing environments. The oxytocin receptor also shows plastic changes during adaptation.

For example, during pregnancy, the activity of oxytocin neurons has been shown to be suppressed. Oxytocin neurons receive long-term inhibitory inputs due to plasma volume expansion and possible hyponatremia during pregnancy. Nitric oxide [153] and allopregnanolone [154] have been shown to inhibit the activity of oxytocin neurons. Inputs of excitatory afferents to oxytocin neurons have also been shown to receive suppressive presynaptic control during pregnancy. Noradrenergic projections from A2 noradrenergic neurons in the nucleus tractus solitarius (NTS) are presynaptically suppressed by the activation of μ-opioid receptors during pregnancy. At the level of axonal terminals in the neurohypophysis, oxytocin release is suppressed by co-released dynorphin. On the other hand, synthesis of oxytocin has been shown to be maintained by relaxin and estrogen, leading to the accumulation of oxytocin so that a large amount of it is released at the appropriate time during delivery [155]. The oxytocin system also shows adaptive changes during lactation. Periodic release of oxytocin during lactation is induced by the synchronous and explosive bursting of oxytocin neurons in the SON and PVN. These bursts appear with long intervals (5–20 min) despite continuous suckling stimuli and are observed only during lactation [156]. This system involves dramatic plastic changes in the SON and PVN during the lactation period [156]. Sucking with skin-to-skin contact facilitates oxytocin release within the brain in both the mother and pups [157].

Oxytocin systems are associated with circadian rhythms. Circulating concentrations of oxytocin rise in the light phase and decline gradually in the dark phase in mice [158]. Oxytocin neurons are activated by food intake, and the number of activated oxytocin neurons per unit amount of food intake (that is, the sensitivity of oxytocin neuron activity in response to food intake) was higher in the light phase than at night-time [159]. The higher oxytocin signaling contributes to a smaller meal size in the inactive light phase of nocturnal mice [63,159].

Oxytocin systems also show adaptive changes during chronic stress. Hypothalamic oxytocin neurons projecting to the dorsal vagal complex have been shown to be more active increased in rats during adaptation to chronic restraint stress, consistent with a view that oxytocin is involved in stress adaptations of the autonomic nervous system [160]. On the other hand, accumulating evidence shows that stressful environments in early life suppress the oxytocin system, leading to long-lasting physiological and behavioral changes [105]. 

Despite the growing interest in allostasis, research is still in its infancy. More work focused on the aspects of allostasis is needed for understanding detailed mechanisms underlying the allostatic effects of oxytocin.

## 4. Resilience and Oxytocin

Oxytocin has also been shown to be associated with increased “resilience” following excessive stress or adversity. Resilience is a complex concept that has been defined from the perspectives of many different research fields, but it can be summarized as the ability to maintain health and adapt positively despite adversity. Resilient persons have a decreased risk of developing cardiovascular diseases and decreased prevalence of major mental illness during their lifespan [161,162]. Resilience is considered to be associated with less reactivity of the HPA axis and greater parasympathetic tone, compared to the status of an individual in stressful conditions [162]. The concept of resilience is used to understand individual differences in stress responses and strategies for coping with stress. Thus, resilience is an important target for prevention of and intervention in stress-related disorders. Although it has been difficult to uncover molecular and neural circuits associated with resilience in research using human subjects, research using animal models has progressed in the past decade and the neurobiological mechanisms of resilience have become clearer. 

In animal models, learned helplessness or repeated social defeat stress, which induce depressive-like behavior, has been used to distinguish between susceptible and resilient animals. Early life stress such as stress from maternal separation and reduced bedding material has also been used in animal models of adversity experienced during childhood and adolescence in humans [163]. Affiliative maternal parenting fosters resilience through its effects on the development and maturation of the brain [164]. The development of resilience requires plasticity in order for organisms to adapt to changing environments and to establish social relationships. Social relationships include social support and belonging to a social group. Oxytocin is involved in the adaptation to changing environments and in the establishment of social relationships, suggesting that oxytocin plays a key role in demonstrating and developing resilience [164].

### 4.1. Roles of Oxytocin in Resilience in Animal Models

Animal studies have shown that oxytocin has persistent effects facilitating resilience in a variety of stress models.

Affiliative tactile stimuli including maternal care and gentle stroking of rats by humans confer resilience against stress [165]. Gentle stroking activates oxytocin neurons in adult rats [166]. Experience of gentle stroking during adolescence enhances the activation of oxytocin neurons in adulthood [167,168]. Social enrichment has been reported to increase plasma oxytocin levels in female rats and to increase novelty-seeking behavior. Telomere length has been shown to be increased by social enrichment via oxytocin signaling, suggesting that oxytocin induced by social enrichment has an anti-aging effect [169]. In oxytocin receptor-deficient mice, an increase in defeat posturing during second social defeat stress by repeated social defeat stress was not observed [11], suggesting that oxytocin facilitates active coping behaviors in response to repeated stressors to confer resilience [170,171]. 

Oxytocin has also been shown to be involved in the effects of early life stress. Oxytocin signaling in the medial prefrontal cortex has been reported to promote stress resilience in response to exposure to predictable maternal separation, while reduction in the expression of the oxytocin receptor has been shown to induce stress susceptibility in response to exposure to unpredictable maternal separation stress [172]. Early-life adversity has been shown to influence vulnerability to drug abuse by weakening oxytocin systems [173]. Early-life adversity disturbs the maturation of oxytocin neural circuits and produces long-lasting weakening of oxytocin systems [105,173]. Neonatal isolation has been reported to reduce partner preference in adult female prairie voles, and the social isolation-induced impairment in partner preference has been shown to be mitigated via pharmacological potentiation of oxytocin release by a melanocortin 3/4 agonist [174]. High oxytocin receptor-binding in the nucleus accumbens (NAc) has been shown to mediate resilience to the effects of daily neonatal isolation stress [174]. Early postnatal administration of an oxytocin receptor agonist has been shown to attenuate microglial inflammation and to normalize myelination, brain connectivity, and cognitive and anxiety-related behaviors in an early life stress model [175]. Oxytocin treatment in rodents has been reported to ameliorate maternal separation-induced behavioral deficits including depressive-like behaviors [176,177] and autistic-like behaviors [178] as well as deficits in social and cognitive memory [179]. Oxytocin administration has also been reported to hinder increases in alcohol drinking (alcohol vulnerability) induced by repeated social defeat during adolescence [180]. The mechanisms underlying the long-lasting effects of oxytocin administration remain unclear. However, oxytocin has been suggested to induce neural growth [181], expression of synaptic proteins [182] and neurogenesis [183]. Oxytocin has also been shown to facilitate the excitatory-to-inhibitory developmental shift of GABAergic neurons [184]. It is possible that these structural and functional effects of oxytocin are involved in its long-lasting actions [105].

Oxytocin has also been shown to be associated with drug vulnerability and alcohol dependence in adulthood. Oxytocin has been reported to reduce drug-abuse vulnerability via the oxytocin receptor in the NAc of adult animals [173]. Pair-housing with familiar mice has been shown to increase plasma oxytocin levels and to prevent social defeat-induced vulnerability to drug abuse [185,186]. Oxytocin injection has been reported to reduce alcohol cue-induced reinstatement response in rats [187]. Oxytocin has also been reported to block increased craving for alcohol in a rat model of alcoholism via the blocking of alcohol’s effects on GABAergic transmission in the central amygdala [188]. 

Oxytocin has also been reported to modulate emotional memory. Reactivated memory becomes temporarily malleable and can integrate new information. Reducing emotional responses before the reactivation of traumatic memories is expected to alleviate PTSD-like symptoms. Single prolonged stress has been shown to induce the PTSD-like symptoms of increased anxiety and reduced arousal in vulnerable rats. In vulnerable rats, oxytocin injection has been shown to activate the prelimbic cortex and basolateral amygdala and to reverse manifestation of PTSD-like behavioral symptoms for a long period [189], suggesting that oxytocin induces emotional remodeling via plastic changes of neural activity.

Oxytocin has also been shown to be involved in individual differences of stress resilience. The resilience of rats in response to isolation-potentiated startle has been shown to be related to distribution patterns of the oxytocin receptor [190]. Distribution of vasopressin receptors and corticotropin-releasing peptide receptors has also been shown to be involved in individual differences [190].

Various animal studies on resilience or vulnerability have shown that oxytocin is important in the process of coping with adversities and recovering from them. Oxytocin also plays an important role in the process of development of resilience during the early-life period by strengthening the affiliative relationship between the mother and littermates.

### 4.2. Roles of Oxytocin in Humans

Early-life adversities such as childhood abuse have also been shown to be associated with lower plasma or CSF oxytocin levels in humans [49,191,192,193]. Female students with high basal oxytocin levels in their saliva were reported to show a positive affect after stress (an emotionally stressful video) and enhanced cognitive-emotional accuracy in a following emotional Stroop task, suggesting that oxytocin buffers negative emotion and facilitates resilience during stress [194]. Maternal depression leads to a higher prevalence of children externalizing and internalizing problems at 10 years after birth due to decreased oxytocin levels in the saliva of the children [195].

Resilience in children who developed chronic PTSD by exposure to war has been reported to be associated with genetic risk variation of the oxytocin receptor gene [196]. People who possess the AA or AG genotype of polymorphic variation (rs2254298) of the oxytocin receptor gene have been shown to be susceptible to the development of mental disorders [197,198], and interaction of the A risk allele of rs2254298 and childhood emotional neglect has been shown to be associated with reduced left-hippocampal volume [199]. Polymorphic variation (rs53576) of the oxytocin receptor gene has been shown to be associated with vulnerability to the effects of early-life adversity [200,201,202] and to also be associated with resilient coping in adulthood, depending on the impact of childhood family environments [203]. The combination of the rs53576 A allele and insecure attachment has been reported to be associated with an increased risk of PTSD in U.S. military veterans [204]. AA homozygotes of rs53576 carriers with exposure to high emotional trauma have been shown to have a smaller volume of the left hippocampus and disruption of the developmental shift of the source of social support from family in early life to friends or peers in adolescence [205]. On the other hand, the GG risk allele of rs53576 was shown to be associated with decreased oxytocin receptor methylation [199]. There are some reports showing a relationship between methylation of the oxytocin receptor gene and resilience in humans [206]. Oxytocin receptor DNA methylation has been shown to be associated with the development of resilience to conduct problems in a study of only children with pre- and post-natal adversity [206]. 

Stress resilience is important for preventing substance use in youth and adults, and the oxytocin system is one of the target areas in treatment for substance-use disorders [207,208]. The expression of oxytocin receptor mRNA and protein in the frontal cortex and striatum has been reported to be increased in humans with an alcohol-use disorder [187]. Oxytocin injection has been reported to reduce activity of the brain circuit related to alcohol cue reactivity in heavy drinkers [187]. In cocaine-dependent men with childhood trauma, oxytocin has been reported to reduce functional magnetic resonance imaging (fMRI) responses in the dorsomedial prefrontal cortex associated with cocaine-seeking behavior and anxiety during withdrawal, and in the amygdala, associated with responses to cocaine cues and craving [209].

Veterans exposed to combat-related trauma have been reported to show higher resting alpha activity in prefrontal regions in magnetoencephalography. The prefrontal cortex subserves working memory and cognitive control, which are disrupted in PTSD. Intranasal oxytocin administration has been reported to normalize alpha activity [210]. Some studies showed the effectiveness of oxytocin administration for amelioration of PTSD symptoms [211,212]. On the other hand, oxytocin administration in subjects who received early life stress has been reported to increase cortisol reactivity and to reduce activity of the limbic system during psychological stress, while oxytocin administration attenuated cortisol reactivity and reduced limbic deactivation in non-stressed subjects. The opposite effects of oxytocin in early life-stressed subjects and non-stressed subjects suggest that oxytocin may enhance stress responses in individuals with early life stress [213].

Research on resilience and oxytocin in humans is still limited. Research has been focused on the alleviation of symptoms and maintenance of mental health following adversity or trauma. Clarifying whether oxytocin is involved in processes that prevent deterioration due to adversity or that foster resilience is a topic for future research.

## 5. Concluding Remarks

**Figure 2:**
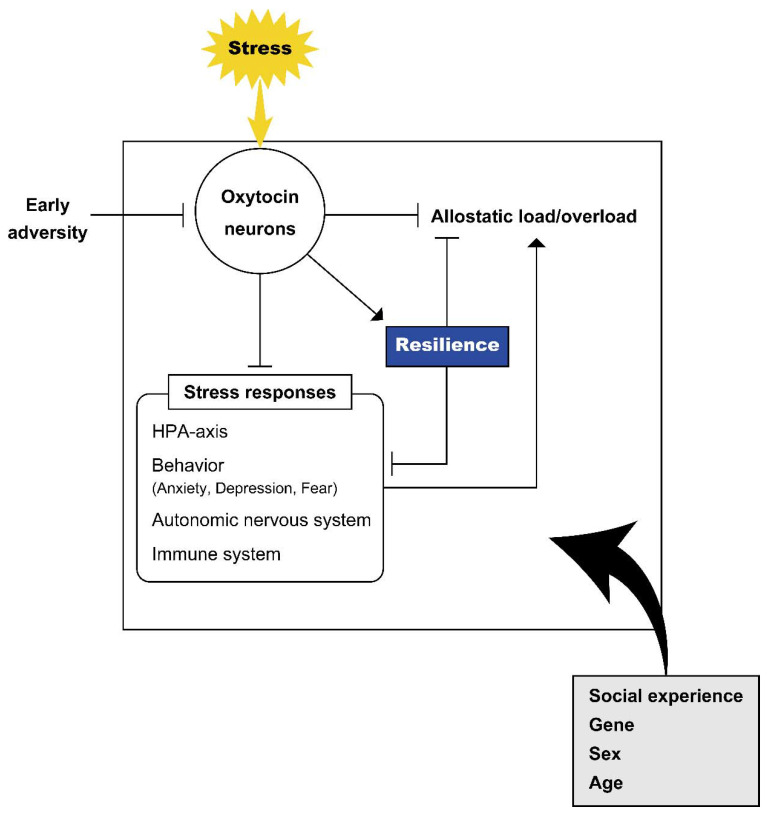
Oxytocin and stress. Schematic representation of the relationship of oxytocin with stress. Stress facilitates activation of oxytocin neurons and release of oxytocin. Various factors including environment and early life adversity plastically modulate activity of the endogenous oxytocin system. Oxytocin influences behavioral and neuroendocrine responses to stress (to maintain homeostasis) and plays a role in the inhibition of allostatic load/overload. Resilience facilitated by oxytocin influences the inhibition of stress responses and allostatic load/overload. Various contexts in individuals including social experience, gene, sex and age modulate the effects of oxytocin and it is possible that these contexts have impacts on the oxytocin system itself, stress responses, allostatic load/overload and resilience, leading to different outcomes.

Stressful stimuli activate oxytocin neurons and promote oxytocin release. Oxytocin modulates stress responses and facilitates adaptation to stress. The effects of oxytocin also depend on the individual situation and environmental context. Oxytocin has been shown to acutely modulate stress responses and to have organizational effects in subjects after early life stress (Figure 2). In this review, we show that oxytocin also plays a role in coping with persistent stress to facilitate allostasis. Allostasis facilitates adaptation not only by changing the internal milieu toward new set points but also by operating other physiological and behavioral systems in response to the changing environment. In fact, organisms show different physiological and behavioral responses in response to a certain stressful stimulus depending on the surrounding social environment. The oxytocin system plays a role in environment-dependent stress responses.

Oxytocin is receiving attention as a target for treatment of stress-related disorders, social disorders, and eating behaviors because oxytocin is considered to be well tolerated and safe for use in adults and children [214,215,216]. However, there are conflicting data on the effects of oxytocin administration. Oxytocin has a high affinity to the oxytocin receptor, but it also has some affinity to vasopressin receptor 1a. Thus, exogenous oxytocin application at a high dosage can activate both the oxytocin receptor and vasopressin receptor 1a, which sometimes has effects opposite to those of the oxytocin receptor alone [144,217,218,219,220]. The proportions of the two receptors that are activated may vary depending on the procedures used in studies, possibly leading to controversial data. Oxytocin injected peripherally has been shown to hardly pass through the blood-brain barrier. Permeability of the blood-brain barrier might depend on the route of oxytocin application, and some studies showed that oxytocin injected intranasally can reach the brain in appropriate amounts or increase central endogenous oxytocin release [221,222,223,224,225], although there are debates concerning the permeability of intranasal oxytocin into the brain [225,226]. Thus, differences in oxytocin administration routes might result in contradictory data. Furthermore, the effects of oxytocin vary depending not only on the dose or route of administration, but also individual factors including genetic variations, gender, age, social environments, and early life experience [225]. Further research to understand the interactions of these factors that influence the effects of oxytocin will enable prediction of risk for stress-related disorders and will advance the clinical use of oxytocin. Oxytocin is cleared rapidly, and its rapid clearance decreases its therapeutic effects. A long-acting oxytocin analogue, which might reach the brain via peripheral injection, has been reported to affect social behaviors [227]. Activation of the endogenous oxytocin system is another strategy. This could be accomplished pharmacologically [228,229,230] or behaviorally [27,28,29,30,231,232,233,234] by, for example, spontaneous pleasurable emotional experiences such as human-dog interactions, massage, gentle touching, physical running and music therapy [235]. In addition, effective activation of the endogenous oxytocin system may strengthen the ability of the individual to adapt and facilitate resilience, leading not only to new therapeutics for or ways for preventing life style-related or early life trauma-related diseases, [236,237] but also health promotion.
